# Determination and Prediction of Standardized Mineral Availabilities in Different Sources of Soybean Meals for Broilers

**DOI:** 10.3390/ani16203251

**Published:** 2026-10-16

**Authors:** Yun Hu, Fanchi Meng, Xin Du, Wenpeng Chen, Liyang Zhang, Xiaoyan Cui, Tingting Li, Shengchen Wang, Chuanlong Wang, Xugang Luo

**Affiliations:** 1 Poultry Mineral Nutrition Laboratory, College of Animal Science and Technology, Yangzhou University, Yangzhou 225000, China; 2 Institute of Animal Science, Chinese Academy of Agricultural Sciences, Beijing 100193, China

**Keywords:** broiler, soybean meal, standardized mineral availability, chemical property, prediction model

## Abstract

This study was conducted to estimate the standardized mineral availabilities (SMAs) of 10 soybean meals (SBMs) fed to broilers, and prediction models were developed and validated based on chemical properties. A total of 308 broilers (22 d of age), having similar initial body weights, were assigned at random to 11 dietary groups, with 7 replicate cages per group and 4 broilers per cage. On d 26, the broilers were given 4 h access to either a mineral-free diet or 10 test SBM-formulated diets. Fecal collection then proceeded for 52 h for SMA measurement. SMA-prediction models were established via stepwise regression using data from 9 SBMs, with the remaining SBM randomly used to validate the models’ accuracy. Soybean meal source influenced (*p* < 0.02) SMAs in broilers. Manganese (Mn) exhibited the lowest standardized availability (SA) at 30.4%, whereas calcium (Ca) showed the highest SA at 66.2%. Six prediction models for SAs of potassium (K), Ca, phosphorus (P), zinc (Zn), Mn, and copper (Cu) in SBMs for broilers were developed from the chemical properties, exhibiting *R*^2^ values spanning 0.487 to 0.964 (*p* < 0.04). The prediction model for standardized Ca availability provided the best goodness-of-fit (*R*^2^ = 0.964, *p* < 0.001). By contrast, this model for standardized Cu availability performed the worst (*R*^2^ = 0.487, *p* = 0.037). Except for Zn and Mn, the predicted SAs for Ca, P, K, and Cu were generally consistent with the measured values. Taken together, the constructed prediction models in our work can serve as a reliable tool for quickly predicting the SAs of Ca, P, K, and Cu in SBMs for broilers.

## Simple Summary

So far, few prediction models have been developed to estimate the standardized mineral availabilities (SMAs) in soybean meals (SBMs) for broilers and other animal species. In this study, the SMAs of SBMs from various sources were assessed in broilers, and subsequently, SMA-prediction models were constructed using chemical properties. Results showed that the SBM source significantly influenced SMAs in broilers, and that most prediction models demonstrated promising accuracy. These outcomes lay the scientific and theoretical groundwork for the rapid prediction of SMAs from chemical properties and the precise formulation of minerals for SBM-based diets in broilers.

## 1. Introduction

Soybean meal (SBM), a by-product generated during soybean oil production, serves predominantly as a protein ingredient for animal feed formulations [1,2]. At present, China faces challenges such as a shortage of protein feed materials, high prices, and heavy reliance on imports, which have driven up production costs in livestock and poultry production. SBMs, as the primary protein sources and accounting for over half of livestock and poultry feed, exhibit considerable variability in quality and nutritional value due to factors including variety, harvest time, soil conditions, processing methods, origin, and production batches [3,4]. These variations significantly influence the overall nutritional composition of feed formulations. In this context, accurately assessing the nutritional values of SBMs and formulating precise diets based on these evaluations can not only help reduce feed costs and resource waste, but also serve as a key factor in mitigating the shortage of protein feed resources for broiler chickens.

Minerals are essential nutrients in broiler diets, participating in various biological processes such as energy storage/utilization, protein metabolism, inflammation, bone metabolism, and immune function [5,6,7]. The background levels of minerals in feed raw materials, particularly microelements, are typically insufficient to meet broiler nutrient requirements [8]. Therefore, mineral supplementation is essential to support broiler growth and production. The current common practice in broiler production is to ignore the background mineral contents of feedstuffs and supplement minerals without adjusting for these basal contributions. This basal-unadjusted mineral supplementation leads to multiple adverse effects: it raises feeding costs, impairs resource use efficiency, and causes environmental contamination through surplus mineral excretion in feces [9]. Consequently, the accuracy of mineral supplementation in broiler diets hinges on a precise assessment of mineral bioavailability in feeds.

Given that apparent mineral availability (AMA) estimates failed to incorporate endogenous mineral losses (EMLs), this omission resulted in a considerably lower estimate of the actual mineral utilization in broiler feeds [10,11]. Hence, it is imperative to adopt an appropriate method to accurately determine and account for EMLs in order to eliminate their interference. Based on measurements of EMLs using mineral-free diets, Nwokolo et al. [12] developed a streamlined approach to estimate the standardized mineral availabilities (SMAs) in SBMs and other protein feedstuffs for 21-day-old white-feathered broilers. However, this method was shown in multiple studies to overestimate the SMAs of feed ingredients in broilers [13,14,15]. This deviation may be attributed to insufficient excreta collection time, leading to incomplete collection and consequently an overestimation of the results [13,14,15]. Therefore, by combining available data from earlier investigations with our own subsequent bench work [13,14,15], our laboratory succeeded in devising a rapid procedure for estimating SMAs that is equally effective for feed ingredients and complete broiler diets [14,16,17]. Employing this newly developed method, we obtained accurate measurements of the SAs for phosphorus (P), calcium (Ca), manganese (Mn), iron (Fe), copper (Cu), and zinc (Zn) in various conventional broiler diets; the method showed good repeatability [14,16,17]. Moreover, the additivity results for SMAs showed that the measured SMA values in corn-soybean meal diets were close to their calculated values based on SMA values in individual feed ingredients (Ca: 48.6% vs. 48.1%; P: 44.9% vs. 46.0%; Mg: 24.9% vs. 22.2%; Cu: 41.7% vs. 39.1%; Fe: 35.3% vs. 33.2%; Mn: 22.7% vs. 22.2%; Zn: 46.2% vs. 48.4%; all *p* ≥ 0.10) [16]. These results further demonstrate that the developed method has good additivity for estimating the SAs of P and other minerals in feed ingredients for white-feathered broiler chickens, and that it can be used to estimate the SAs of macro- and trace minerals, including Ca, P, Mg, Cu, Fe, Mn, and Zn. Recently, Du et al. [18] evaluated SMAs across 10 different sources of corn for chickens and successfully developed SMA-prediction equations from the chemical properties of these corns. Our previous work also determined the SMAs from 10 different varieties of rapeseed meals for broilers and developed SMA-prediction equations on the basis of their chemical properties [19]. However, limited studies have been published on the development of prediction models for SMAs in SBMs for broilers or other animals.

Thus, the hypothesis of this study was that the chemical properties of SBMs could serve as a basis for developing prediction models for SMAs in broilers. To evaluate this hypothesis, this study initially analyzed the chemical properties and mineral contents in 10 SBMs. Subsequently, their SMAs were quantified in broilers, and these values were used to establish prediction models derived from the SBMs’ chemical properties. Finally, these prediction models were validated for accuracy.

## 2. Materials and Methods

### 2.1. Ethical Statement

All in vivo experiments performed in this research were reviewed and approved by the Yangzhou University Laboratory Animal Management Committee (Ethical approval No. 202107001; approval date: 7 July 2021).

### 2.2. Experimental Design and Treatments

This experiment adopted a completely randomized design and comprised 11 dietary treatments: 1 mineral-free diet control and 10 SBM-based test diets.

### 2.3. Soybean Meals, Animals and Diets

Ten tested SBMs (numbered SBM1 to SBM10) were obtained from various distributors nationwide and imported mainly from the United States and Brazil. The distributors were unable to provide complete traceability information, including geographic regions, processing plants, suppliers, and production batches; their specific origins are detailed in Table 1.

The experiment used 352 male Arbor Acres broilers, all at 1 d of age, supplied by Beijing Huadu Breeding Co., Ltd. (Beijing, China). They were kept in cages with electric heating and automatic temperature regulation, which were made of plastic-coated steel and measured 100 × 50 × 45 cm. Fibreglass feeding troughs and water nipples were installed in each cage. During d 1–21, all birds were given ad libitum access to a single complete micro-pelleted ration composed of corn and SBM, which was obtained from Zhengda Feed Co., Ltd. (Beijing, China). On the morning of d 22 (8:00 A.M.), a total of 308 broilers with an average body weight of 1.10 kg were randomly distributed among 11 treatment groups, with 7 replicate cages per group and 4 birds per replicate cage. Subsequently, the cloaca of each bird was secured by suturing and immobilized with hollow plastic bottle caps [14,16]. Thereafter, all broilers were subjected to a 62 h recovery and acclimation period and received the same diet as that offered from d 1 to d 21. On d 24 at 22:00, an 8 h fast was imposed on all broilers by removing feed. On d 25 at 6:00, the broilers first received a mineral-free diet for 4 h and were then fasted for 24 h to ensure their gastrointestinal tracts were emptied [14,16,17,20]. At 10:00 on d 26, bottle caps were equipped with fecal collection bags for all broilers. Immediately afterward, the birds received either the pre-weighed mineral-free diet or 1 of the 10 SBM diets, with access lasting 4 h. Any feed left was subsequently weighed. A continuous 52 h fecal collection was conducted starting at the time of feed provision. All broilers were given unlimited access to deionized water. Meanwhile, the lighting regime was set to constant illumination over the whole trial.

A mineral-free diet was prepared according to Liu et al. [16], containing soybean oil, dextrose, gelatin, and synthetic AAs as the primary sources of energy, protein, and AAs. To maintain consistency with the mineral-free diet in terms of metabolizable energy (ME), crude protein (CP), and major amino acid profiles, 10 experimental SBM-based diets were formulated. In these diets, the test SBMs replaced glucose, soybean oil, or synthetic amino acids respectively. With the exception of mineral contents, the experimental diets were designed to meet or exceed the nutritional requirements for broilers aged 22 to 42 d during the growing phase, in accordance with the recommendations of the Feeding Standard of Chicken in China (2004) [21]. The chemical properties of the 10 tested SBMs are presented in Table 2, while Table 3 outlines the ingredient composition and nutrient levels for the mineral-free diet and the 10 experimental SBM-based diets. The broilers were fed all these diets in mash form.

### 2.4. Sample Collections and Preparations

All samples (both SBMs and diets) were collected and subjected to chemical analysis. For SBMs, the assay covered conventional nutrients, anti-nutrients, and minerals including potassium (K), P, Ca, magnesium (Mg), Mn, Fe, Zn, and Cu. For diets, the assay focused on CP and the same set of minerals. As the fecal collection bags were typically filled within about 4 h, excreta samples were collected at that same interval. The AMAs and SMAs were derived from the 4 h feed intake values and the weights of dried excreta gathered from the broilers over 52 h, both of which are listed in Table 4. Fecal samples from 4 chickens per replicate cage were pooled to form a composite sample. After drying at 65 °C in a forced-air oven, the composite samples were analyzed for K, Ca, Mg, P, Zn, Mn, Fe, and Cu, with the analytical outcomes compiled in Table 5. All samples (SBMs, diets, and dried excreta) were subjected to grinding and subsequent sieving through a 0.45 mm sieve before analysis.

### 2.5. Determination Indicators and Methods

The contents of moisture (method 934.01), CP (method 976.05), CF (method 978.10), neutral detergent fiber (NDF, method 2002.04), crude ash (CA, method 942.05), acid detergent fiber (ADF, 973.18), and ether extract (EE, method 954.02) were analyzed according to the Association of Official Agricultural Chemists (AOAC) international [22]. The contents of phytic acid (PA; SAP-2-Y, Suzhou, China) and trypsin inhibitor (TI; MM-1613O2, Yancheng, China) in SBM samples were quantified using commercial kits. Quantification of K, Ca, Mg, P, Zn, Mn, Fe, and Cu in SBMs, diets, and dried excreta was carried out according to the analytical protocol of Hu et al. [19]. All samples were analyzed in duplicate.

### 2.6. Calculations

Values for the AMA, SMA and EML were derived from the equations below:

(1) $$\mathrm{AMA}\left(\%\right)=\frac{M_{i}-M_{o}}{M_{i}}\times 100$$

EML(mg/chicken) = M_c_ × M_e_ (2)

(3) $$\mathrm{SMA}\left(\%\right)=\frac{M_{i}-{(M}_{o}-\mathrm{EML})}{M_{i}}\times 100$$

where M_i_ is defined as the total mineral consumed (mg/bird) from the tested diet, whereas M_o_ corresponds to the total mineral excreted (mg/bird) by birds via the droppings. Correspondingly, M_c_ and M_e_ represent the mineral concentration in the excreta (mg/g) and the total excreta production (g/bird), respectively, for birds on the mineral-free diet.

### 2.7. Statistical Analyses

Statistical analyses of AMA and SMA data were conducted using one-way ANOVA within the GLM procedure of SAS 9.4. Treatment differences were evaluated using the LSD method. Subsequently, Pearson correlation analysis between the chemical properties and SMAs of SBMs was performed using SAS. The SMA-prediction models were developed using stepwise regression based on the chemical properties of 9 SBM samples (SBM1 to SBM9). The accuracy of the SMA-prediction models was then validated using an additional SBM sample (SBM10), which was generated by the RANDGEN subroutine in SAS. The replicate cage served as the basic unit of analysis. Statistical significance was set at a threshold of *p* < 0.05.

## 3. Results

### 3.1. Chemical Properties and Mineral Contents of SBMs

The chemical analysis results for the SBM samples are compiled in Table 2. A wide range of variation was observed in the chemical compositions and mineral contents of SBMs. The contents of moisture, CP, EE, CF, NDF, ADF, CA, NFE, PA, and TI ranged from 8.9% to 13.4%, 43.2% to 47.1%, 0.79% to 1.81%, 4.65% to 9.60%, 9.08% to 19.55%, 4.69% to 12.10%, 6.05% to 7.60%, 24.1% to 30.1%, 7.62% to 13.80%, and 11.31% to 17.13%, respectively. The CVs above 10.0% were observed for all chemical properties other than CP and NFE. Notably, the lowest CV was 2.76% for CP and the highest CV was 30.4% for ADF. Among the macroelements, Ca varied from 0.220% to 0.363%, P from 0.384% to 0.697%, K from 2.20% to 2.39%, and Mg from 0.319% to 0.361%. Among them, Ca and P showed relatively high CVs, reaching 17.2% and 23.3% respectively, while K and Mg exhibited relatively low CVs of 2.80% and 3.69% respectively. Among the microelements, Cu varied from 7.6 mg/kg to 14.2 mg/kg, Fe from 76 mg/kg to 302 mg/kg, Mn from 19.9 mg/kg to 35.3 mg/kg, and Zn from 28.4 mg/kg to 48.3 mg/kg. The CVs for these four microelements are relatively high, with values of 18.6% for Cu, 44.8% for Fe, 23.0% for Mn, and 20.4% for Zn.

### 3.2. AMAs of SBMs for Broilers

Table 6 shows that the SBM source influenced (*p* < 0.004) the apparent availability (AA) values of Ca, P, K, Mg, Cu, Fe, Mn and Zn in broilers. Regarding the macroelements, the AA of Ca spanned 36.4% to 54.1%, whereas P AA varied from −26.4% to 3.3%. The corresponding ranges for K and Mg AAs were 6.7% to 25.6% and 12.5% to 24.2%, respectively. Their corresponding CVs were 12.4% for Ca, 186% for P, 28.8% for K, and 22.1% for Mg. For the four tested trace elements, the AA of Cu ranged from 4.3% to 34.7%, whereas the AA of Fe varied more widely, between −22.6% and 33.1%. For Mn and Zn, the respective AAs varied from −9.4% to 18.0% for Mn and from 9.4% to 42.7% for Zn. Their corresponding CVs were 43.9% for Cu, 224.5% for Fe, 115.4% for Mn, and 28.5% for Zn. Among the aforementioned mineral elements, the average AMA values were the lowest for P (−5.8%) and the highest for Ca (48.3%).

### 3.3. SMAs of SBMs for Broilers

Table 7 shows that the SBM source influenced (*p* < 0.02) the SAs of Ca, P, K, Mg, Cu, Fe, Mn and Zn in broilers. Regarding the macroelements, the SAs spanned 56.4% to 69.8% for Ca, 40.3% to 68.1% for P, 34.0% to 51.1% for K, and 34.3% to 48.7% for Mg. The corresponding CVs were observed at 7.4% for Ca, 16.0% for P, 11.0% for K, and 11.1% for Mg. For microelements, the SAs of Cu, Fe, Mn, and Zn ranged from 33.2% to 58.0%, 12.4% to 58.0%, 16.9% to 39.1%, and 23.1% to 53.7%, with corresponding CVs of 16.6%, 39.3%, 21.4%, and 20.2%, respectively. Among the aforementioned mineral elements, the average SMA values were the lowest for Mn (30.4%) and the highest for Ca (66.2%).

### 3.4. Correlation Analyses

According to Table 8, the SAs of Ca, P, K, Mn, and Zn showed a negative correlation (*p* < 0.05) with PA contents in SBMs. Similarly, negative associations (*p* < 0.05) with ADF were restricted to the SAs of Ca, P, and K, whereas the only K SA was negatively correlated (*p* < 0.05) with CF. In contrast, a positive correlation (*p* < 0.05) was observed between CP contents and the SAs of Ca, P, and Cu.

### 3.5. Prediction Models for SMAs in SBMs for Broilers

As shown in Table 9, 6 SMA-prediction models were successfully developed for SBMs in broilers, exhibiting *R*^2^ values from 0.487 to 0.964 (*p* < 0.04), using their chemical properties. Specifically, standardized Ca availability was predicted using CA and PA; standardized P availability was predicted by CF, CA, and PA; standardized K availability was estimated via NDF and ADF; standardized Cu availability was predicted using CP; standardized Mn availability was estimated by PA; and standardized Zn availability was predicted using PA and NFE. While stepwise regression models for standardized Ca and P availabilities achieved excellent fits (*R*^2^ > 0.90, *p* < 0.001), the model for Cu availability performed less well, with the *R*^2^ of only 0.487 (*p* = 0.037).

### 3.6. Validation of the SMA-Prediction Models for SBMs in Broilers

As shown in Table 10, a single SBM sample (SBM10) was randomly selected for validation to provide a preliminary assessment of the developed models. The absolute differences between these predicted and measured SMA values were 6.6% (Ca), 6.7% (P), 0.2% (K), 6.7% (Cu), 12.2% (Mn), and 16.7% (Zn). With the exception of Mn and Zn, which exhibited comparatively large discrepancies, the predicted SAs for the remaining elements were all within the range of the measured values ± their respective standard deviations. Consequently, the prediction models demonstrate strong predictive performance, yielding reliable outcomes for most of the tested elements.

## 4. Discussion

In this study, the chemical properties of SBM samples from various origins were characterized, and their SAs of Ca, K, P, Mg, Mn, Zn, Fe, and Cu for broilers were evaluated. Furthermore, SMA-prediction models were established, with their predictive accuracy subsequently validated. The findings revealed that, with the exception of Mn and Zn, the chemical properties of the SBM samples (including PA, CA, CF, CP, NFE, NDF, and ADF) could reliably support the development of SMA-prediction models for broilers, a result that supports our hypothesis. These results, which have not been documented in previous reports, offer a scientific and theoretical foundation for both rapid prediction of SMAs from chemical properties and precise mineral nutrition in SBM-based broiler diets.

The present study revealed marked variation in the chemical properties of SBM samples derived from 10 distinct origins. All chemical properties, except for CP, NFE, K, and Mg, exhibited CVs greater than 10%. These disparities stem from a range of variables, including processing techniques, husk separation levels, varietal traits, and climatic conditions [23,24]. Li et al. [24] evaluated SBM samples obtained from the United States, Argentina, Brazil, and China and found that the mean levels of NDF, ADF, Ca, and P were consistent with those determined in the current work, while the EE and CA concentrations were lower and the CP concentration was higher. This discrepancy may be due to differences in origin and processing methods of SBMs. Overall, the selected SBM samples showed substantial variation in their chemical properties, confirming that they are well-suited to represent different provenances.

When EMLs were not deducted, the measured AMAs were generally low and occasionally negative. For example, Liu et al. [16] reported that the apparent availability values of Ca, P, Cu, Fe, Mn, and Zn in SBM were 21.0%, −21.9%, 14.9%, 9.4%, 14.2%, and 35.3%, respectively. Moreover, we previously reported that the apparent availability values of Ca, P, K, Mg, Cu, Fe, Mn, and Zn in rapeseed meals were 11.1%, −30.9%, 38.0%, −1.71%, −44.0%, −10.8%, −0.08%, and −0.7%, respectively [19]. Hence, compared with AMAs [11,25], SMA values determined in this study more accurately reflect their utilization. However, few studies have investigated the SMAs in SBMs for broilers. Liu et al. [17] determined the SMAs of SBM for 24-day-old broilers to be 51.1% for Ca, 50.2% for P, 36.3% for Cu, 30.8% for Mn, and 49.7% for Zn. In a follow-up experiment with 28-day-old broilers, Liu et al. [16] reported SAs for Ca, P, Cu, Fe, Mn, and Zn in SBM, with corresponding values of 54.3%, 51.1%, 39.7%, 27.6%, 26.1%, and 51.0%. The present study demonstrated that the SAs of Ca, P, Mg, K, Cu, Fe, Mn, and Zn in broilers were significantly affected by SBM source. Among them, the SAs of P, Mn, and Zn aligned with previously reported values, whereas those of Ca, Cu, and Fe were higher. The compositional heterogeneity of SBMs likely explained the observed differences. In contrast to Liu et al. [16,17], who relied on a single SBM, our investigation examined 10 distinct SBMs displaying marked variations in their chemical properties. Moreover, this study found that the SAs for K and Mg in SBMs were 45.0% and 42.2%, respectively. Previously, we determined the SAs of Ca, P, K, Mg, Cu, Fe, Mn, and Zn in rapeseed meals for broilers, which were 29.5%, 25.2%, 25.4%, 20.2%, 35.3%, 14.3%, 12.9%, and 16.7%, respectively [19]. Compared with rapeseed meals, SBMs provided significantly higher SMAs for broilers. It should be pointed out that the WPSA protocol measures P digestibility at the terminal ileum using an indigestible marker, a low-P basal diet, and at least two supplemental levels of the test P source, with precaecal digestible phosphorus (pcdP) estimated from the slope of linear regressions [26]. In contrast, our SMA procedure estimates standardized total-tract mineral availability, based on total excreta collection and correction for basal endogenous mineral losses measured with a mineral-free diet. Therefore, the SMA approach differs from the WPSA pcdP protocol in several fundamental aspects, such as the measurement site (total tract vs. terminal ileum), the method (total excreta collection vs. ileal digesta collection with an indigestible marker), and the way in which endogenous losses are measured (the mineral-free diet vs. regression-based estimation). Because of these differences, SMA and pcdP values should not be directly compared numerically. Biologically, SMA values represent the proportion of dietary minerals that are absorbed and utilized after removing basal endogenous losses. They are intended for practical diet formulation, in which additivity of ingredient values is essential. In addition, we have demonstrated that our new method has good additivity and repeatability for estimating the standardized availabilities of P and other major minerals in feed ingredients for white-feathered broiler chickens. Hence, the SMA approach is complementary to, but not equivalent to, the WPSA pcdP protocol, and our values are not numerically identical to WPSA pcdP values.

Constructing prediction models for SMAs based on the chemical properties of SBMs is key for achieving rapid prediction of SMAs in SBMs for broilers. Adopting this method helps to improve the accuracy of mineral supplementation in SBM-based diets while keeping mineral excretion to a minimum in broiler production. Fiber, TI, and PA are the main anti-nutritional factors in SBM, as they can impair the digestion and absorption of nutrients in animals, thereby reducing growth performance [27]. PA can inhibit enzyme activity in the small intestine and stomach of animals and bind to metal ions to form complexes, thereby affecting the utilization of mineral elements [28]. TI can reduce the activity of trypsin in animals, affecting protein digestion and utilization [29]. Insoluble fiber can accelerate intestinal emptying, shorten the contact time between chyme and the intestinal wall, and thus reduce the absorption rate of mineral elements. In this study, all SMAs were negatively correlated with the contents of CF, ADF, and NDF, and positively correlated with CP content. Except for the SAs of Mn and Cu, all other SMAs were negatively correlated with TI and PA contents. This indicates that the anti-nutritional factor content in SBM can affect the absorption and utilization of mineral elements in broilers. Furthermore, 6 SMA-prediction models in SBMs for broilers were built based on the contents of PA, CF, ADF, NDF, CA, CP, and NFE as primary predictive factors. Currently, there is relatively little research on prediction models for SAMs in broilers. Du et al. [18] developed 7 SMA-prediction models in corn for broilers on the basis of their chemical properties. It was revealed that the SAs of Ca, P, Mg, K, and Zn were negatively correlated with the contents of PA, ADF, and CF, but positively correlated with CP [18]. We previously developed 8 SMA-prediction models in rapeseed meals for broilers on the basis of their chemical properties [19]. Consistently, these prediction models also revealed a negative correlation between NDF or ADF contents and the SAs of Cu, Fe, and Mn. Similar to previous reports, we found that the prediction models for the SAs of Ca, P, Mn, and Zn were negatively associated with PA, while those for K were negatively associated with NDF and ADF, and those for Cu were positively associated with CP. Taken together, these findings highlight that the contents of PA, CP, ADF, and NDF are promising predictors that could be employed to establish SMA-prediction models in SBMs for broilers.

Subsequently, to evaluate the predictive performance of the established SMA-prediction models, we randomly selected SBM10 as the validation sample. With the exception of Mn and Zn, the predicted standardized availability values of Ca, P, K, and Cu were largely aligned with the determined values. Nonetheless, because the models were developed using only 9 samples and stepwise selection, they were exploratory and at high risk of overfitting. The apparent *R*^2^ values are likely optimistic, and the regression coefficients may be unstable. Only one independent SBM sample is insufficient for external validation. Therefore, a further external validation using a larger, independent dataset that includes a wider range of SBMs is required before these equations can be used to predict the SIDs of minerals in SBMs in commercial broiler operations.

## 5. Conclusions

Six SMA-prediction models for SBMs were successfully developed in broilers in this study. Except for the Mn and Zn SA prediction models, the remaining prediction models for Ca, P, K, and Cu SAs delivered reliable results. These prediction models offer a practical reference for rapidly estimating SMAs in SBMs for broilers on the basis of their chemical properties (PA, CF, ADF, NDF, CA, CP, and NFE).

## Figures and Tables

**Table 1:** The sources of 10 different tested SBMs.

Number	Sources	Number	Sources
SBM1	Brazil	SBM6	Brazil
SBM2	USA	SBM7	Brazil
SBM3	USA	SBM8	China
SBM4	Brazil	SBM9	USA
SBM5	USA	SBM10	USA

SBM, soybean meal.

**Table 2:** The contents of conventional nutritional components, anti-nutritional factors, and mineral elements in 10 tested SBMs (air-dry basis) ^1^.

Items (%)	SBM Numbers										Mean	SD	CV (%)
	SBM1	SBM2	SBM3	SBM4	SBM5	SBM6	SBM7	SBM8	SBM9	SBM10			
Moisture	11.2	11.3	12.6	13.0	13.4	12.2	12.8	12.1	8.9	10.1	11.8	1.4	11.9
CP	45.1	44.6	46.7	43.2	44.7	46.5	45.3	46.8	46.3	47.1	45.6	1.3	2.8
EE	1.25	1.61	1.46	1.36	1.81	1.37	1.52	1.22	0.92	0.79	1.33	0.31	23.0
CF	7.14	6.31	6.72	9.60	6.44	4.65	7.5	8.46	6.7	5.78	6.93	1.38	19.9
NDF	13.8	14.0	10.8	19.6	16.3	12.8	16.5	10.6	10.7	9.1	13.4	3.3	24.4
ADF	7.13	8.66	5.46	12.10	7.88	5.59	8.38	6.56	5.54	4.69	7.20	2.19	30.4
CA	6.13	6.08	6.31	6.05	6.15	7.37	7.60	7.33	7.57	7.35	6.79	0.69	10.2
NFE	29.2	30.1	26.2	26.9	27.5	27.9	25.3	24.1	29.6	28.9	27.6	1.9	6.88
PA (g/kg)	7.62	13.44	8.06	13.30	8.84	9.73	9.98	9.07	11.41	13.8	10.53	2.31	22.0
TI (g/kg)	11.4	17.1	14.8	12.4	11.3	14.8	15.5	13.4	16.0	12.7	13.9	2.0	14.4
Ca	0.232	0.274	0.258	0.220	0.340	0.252	0.283	0.270	0.339	0.363	0.283	0.048	17.1
P	0.446	0.384	0.443	0.397	0.663	0.501	0.471	0.418	0.697	0.651	0.507	0.118	23.3
K	2.20	2.29	2.32	2.23	2.29	2.38	2.39	2.39	2.37	2.37	2.34	0.06	2.40
Mg	0.338	0.319	0.346	0.359	0.361	0.328	0.333	0.335	0.348	0.347	0.341	0.013	3.89
Cu (mg/kg)	8.9	11.6	10.7	7.6	12.3	12.5	12.4	11.5	14.1	14.2	11.6	2.2	18.6
Fe (mg/kg)	144	76	187	145	115	113	114	88	302	183	147	66	44.8
Mn (mg/kg)	21.3	22.5	23.6	19.9	32.1	35.1	32.8	22.4	34.1	35.3	27.90	6.42	23.0
Zn (mg/kg)	36.0	28.4	32.0	31.5	48.3	37.1	34.7	30.1	48.0	46.7	37.3	7.62	20.4

^1^ Values determined by analysis; each value is based on triplicate determinations. SBM, soybean meal; CP, crude protein; EE, ether extract; CF, crude fiber; NDF, neutral detergent fiber; ADF, acid detergent fiber; CA, crude ash; NFE, nitrogen-free extract; PA, phytic acid; TI, trypsin inhibitor; Ca, calcium; P, phosphorus; Mg, magnesium; K, potassium; Cu, copper; Fe, iron; Mn, manganese; Zn, zinc; SD, standard deviation; CV, coefficient of variation.

**Table 3:** Composition and nutrient levels of mineral-free diet and 10 tested SBM diets for broilers (as-fed basis).

Item	Mineral-Free Diet	SBM1 Diet	SBM2 Diet	SBM3 Diet	SBM4 Diet	SBM5 Diet	SBM6 Diet	SBM7 Diet	SBM8 Diet	SBM9 Diet	SBM10 Diet
Ingredients (%)											
Soybean meal	—	42.34	42.77	40.85	44.27	42.70	41.00	42.14	40.76	41.24	40.50
Dextrose ^1,@^	72.79	52.40	51.93	54.06	50.27	52.01	53.90	52.63	54.14	53.63	54.44
Gelatin ^1,#^	17.13	—	—	—	—	—	—	—	—	—	—
Soybean oil ^1^	4.00	4.24	4.29	4.06	4.47	4.28	4.07	4.21	4.06	4.10	4.02
Cellulose ^2,&^	2.00	0.67	0.67	0.67	0.67	0.67	0.67	0.67	0.67	0.67	0.67
L-Lys HCL ^2,&^	0.37	—	—	—	—	—	—	—	—	—	—
DL-Met ^2,&^	0.57	0.20	0.19	0.21	0.17	0.19	0.21	0.20	0.22	0.21	0.22
L-Thr ^2,&^	0.45	—	—	—	—	—	—	—	—	—	—
L-Trp ^2,&^	0.17	—	—	—	—	—	—	—	—	—	—
L-Ile ^2,&^	0.54	—	—	—	—	—	—	—	—	—	—
L-Leu ^2,&^	0.66	—	—	—	—	—	—	—	—	—	—
L-Val ^2,&^	0.43	—	—	—	—	—	—	—	—	—	—
L-Phe ^2,&^	0.74	—	—	—	—	—	—	—	—	—	—
L-Arg ^2,&^	0.15	0.15	0.15	0.15	0.15	0.15	0.15	0.15	0.15	0.15	0.15
Vitamin premix ^3^	100.00	100.00	100.00	100.00	100.00	100.00	100.00	100.00	100.00	100.00	100.00
Total	4.00	4.24	4.29	4.06	4.47	4.28	4.07	4.21	4.06	4.10	4.02
Nutrient levels											
ME ^4^ (kcal/kg)	2981	2981	2981	2981	2981	2981	2981	2981	2981	2981	2981
CP ^4^ (%)	19.20	19.20	19.20	19.20	19.20	19.20	19.20	19.20	19.20	19.20	19.20
Met ^4^ (%)	0.70	0.45	0.44	0.45	0.43	0.44	0.45	0.45	0.46	0.45	0.46
Lys ^4^ (%)	1.01	1.13	1.15	1.09	1.19	1.14	1.10	1.13	1.09	1.11	1.09
Met + Cys ^4^ (%)	0.72	0.72	0.72	0.72	0.72	0.72	0.72	0.72	0.72	0.72	0.72
Ca ^5^ (%)	0.00	0.15	0.15	0.17	0.16	0.16	0.17	0.16	0.14	0.14	0.16
P ^5^ (%)	0.00	0.25	0.27	0.27	0.25	0.28	0.25	0.27	0.25	0.26	0.25
K ^5^ (%)	0.00	0.97	1.04	1.05	1.02	1.07	1.05	1.08	0.99	1.03	1.04
Mg ^5^ (%)	0.00	0.14	0.16	0.14	0.16	0.16	0.14	0.14	0.13	0.15	0.15
Cu ^5^ (mg/kg)	0.26	5.18	4.89	6.37	4.98	5.04	6.70	6.72	5.20	5.05	5.67
Fe ^5^ (mg/kg)	8.05	79.85	84.71	123.71	104.79	74.16	84.46	73.65	61.44	64.39	85.57
Mn ^5^ (mg/kg)	0.34	12.19	12.83	15.82	12.77	13.28	16.74	18.63	13.77	12.43	13.55
Zn ^5^ (mg/kg)	0.08	19.03	19.76	21.22	20.56	20.10	20.15	19.90	18.48	17.36	17.72

^1^ Food grade. ^2^ Reagent grade. ^3^ For the grower diet, provided per kilogram of diet: vitamin A (all-trans-retinol acetate), 8000 IU; cholecalciferol, 3000 IU; vitamin E (all-rac-α-tocopherol acetate), 22 IU; vitamin K (menadione sodium bisulfate), 2 mg; vitamin B_1_ (thiamine mononitrate) 2 mg; vitamin B_2_ 6.4 mg; vitamin B_6_ 3 mg; vitamin B_12_ 0.02 mg; calcium pantothenate 10 mg; niacin 36 mg; folate 1 mg; biotin 0.1 mg; choline (choline chloride) 500 mg. ^@^ Purchased from Shandong Xiwang Pharmaceutical Co., Binzhou, China. ^#^ Purchased from Jiangxi Fumitai Biotechnology Co., Jiujiang, China. ^&^ Purchased from Shanghai Yuanjiu Biotechnology Co., Shanghai, China. ^4^ Calculated values. ^5^ Determined by analysis and each value is based on triplicate determinations.

**Table 4:** Feed intakes and dry excreta weights of broilers in each treatment group ^1^.

Group Numbers	Feed Intakes (g/Bird. 4 h)	Dry Excreta Weights (g/Bird. 52 h)
Mineral-free diet	30.5 ± 5.7	5.6 ± 0.9
SBM1 diet	53.6 ± 3.3	12.9 ± 1.3
SBM2 diet	48.6 ± 6.8	12.6 ± 1.5
SBM3 diet	48.5 ± 4.1	11.5 ± 1.0
SBM4 diet	48.4 ± 6.5	12.8 ± 1.1
SBM5 diet	50.3 ± 4.4	12.3 ± 0.8
SBM6 diet	50.2 ± 4.0	12.8 ± 1.1
SBM7 diet	50.6 ± 5.1	12.7 ± 1.2
SBM8 diet	54.2 ± 3.2	12.1 ± 0.7
SBM9 diet	54.3 ± 6.3	12.9 ± 0.9
SBM10 diet	46.1 ± 4.1	11.9 ± 0.4

^1^ Values represent the means ± standard deviations (SDs) of 5–7 replicate cages (4 birds per replicate cage) (*n* = 5–7). SBM, soybean meal.

**Table 5:** Mineral element contents in the dry excreta of broilers in each treatment group ^1^.

Group Numbers	Ca (%)	P (%)	Mg (%)	K (%)	Cu (mg/kg)	Fe (mg/kg)	Mn (mg/kg)	Zn (mg/kg)
Mineral-free diet	0.248 ± 0.06	1.48 ± 0.21	0.296 ± 0.03	2.40 ± 0.33	12.8 ± 3.4	222 ± 44	28.2 ± 6.9	22.6 ± 5.9
SBM1 diet	0.306 ± 0.03	1.03 ± 0.08	0.475 ± 0.03	3.18 ± 0.20	18.6 ± 2.8	323 ± 38	48.0 ± 5.3	57.0 ± 7.6
SBM2 diet	0.365 ± 0.05	1.19 ± 0.11	0.475 ± 0.04	3.45 ± 0.45	18.4 ± 2.2	351 ± 36	53.9 ± 2.6	68.9 ± 7.5
SBM3 diet	0.345 ± 0.02	1.18 ± 0.06	0.540 ± 0.04	3.64 ± 0.13	17.5 ± 1.4	332 ± 52	54.5 ± 5.7	59.5 ± 5.9
SBM4 diet	0.364 ± 0.06	1.21 ± 0.10	0.519 ± 0.05	3.61 ± 0.41	16.6 ± 3.0	405 ± 29	50.5 ± 7.6	57.7 ± 9.5
SBM5 diet	0.318 ± 0.03	1.14 ± 0.12	0.500 ± 0.02	3.44 ± 0.36	16.8 ± 1.3	341 ± 91	49.2 ± 2.7	48.5 ± 4.4
SBM6 diet	0.322 ± 0.03	0.96 ± 0.07	0.436 ± 0.02	3.05 ± 0.20	18.5 ± 1.8	291 ± 25	59.2 ± 4.1	50.2 ± 6.6
SBM7 diet	0.316 ± 0.04	1.06 ± 0.10	0.457 ± 0.03	3.44 ± 0.39	19.7 ± 3.3	254 ± 27	61.0 ± 7.4	49.4 ± 6.6
SBM8 diet	0.311 ± 0.05	1.10 ± 0.09	0.462 ± 0.02	3.50 ± 0.21	17.5 ± 1.8	241 ± 19	54.7 ± 7.6	49.3 ± 7.4
SBM9 diet	0.334 ± 0.08	1.07 ± 0.07	0.471 ± 0.02	3.38 ± 0.20	16.9 ± 2.8	342 ± 18	50.9 ± 7.3	49.0 ± 4.8
SBM10 diet	0.327 ± 0.04	1.10 ± 0.07	0.428 ± 0.02	3.15 ± 0.21	17.6 ± 1.7	242 ± 16	48.7 ± 4.7	45.3 ± 6.9

^1^ Values represent the means ± standard deviations (SDs) of 5–7 replicate cages (4 birds per replicate cage) (*n* = 5–7). SBM, soybean meal; Ca, calcium; P, phosphorus; Mg, magnesium; K, potassium; Cu, copper; Fe, iron; Mn, manganese; Zn, zinc.

**Table 6:** AMAs of different sources of SBMs in broilers ^1^.

Items (%)	SBM Numbers										Mean	Pooled SE	CV (%)	*p*-Value
	SBM1	SBM2	SBM3	SBM4	SBM5	SBM6	SBM7	SBM8	SBM9	SBM10				
Ca AA	49.5 ^a^	36.4 ^b^	54.1 ^a^	38.8 ^b^	51.4 ^a^	53.1 ^a^	52.0 ^a^	51.4 ^a^	48.2 ^a^	49.8 ^a^	48.3	1.0	12.4	<0.001
P AA	−0.4 ^a^	−23.1 ^b^	−1.0 ^a^	−26.4 ^b^	−1.4 ^a^	3.3 ^a^	2.0 ^a^	2.1 ^a^	0.4 ^a^	−15.8 ^b^	−5.8	1.8	186.6	<0.001
K AA	21.1 ^ab^	13.3 ^bc^	17.0 ^ab^	6.7 ^c^	23.7 ^a^	25.6 ^a^	19.9 ^ab^	21.0 ^ab^	21.5 ^ab^	21.2 ^ab^	19.0	1.1	28.8	0.003
Mg AA	16.3 ^bc^	21.5 ^ab^	12.5 ^c^	12.9 ^c^	24.2 ^a^	21.3 ^ab^	19.6 ^ab^	23.3 ^a^	22.1 ^ab^	23.6 ^a^	19.9	0.8	22.1	<0.001
Cu AA	13.0 ^cd^	4.3 ^e^	34.7 ^a^	12.4 ^de^	18.1 ^cd^	29.2 ^ab^	26.6 ^abc^	24.9 ^bc^	26.1 ^abc^	19.6 ^cd^	21.3	1.4	43.9	<0.001
Fe AA	3.2 ^c^	−3.0 ^c^	33.1 ^a^	−2.8 ^c^	−0.8 ^c^	11.8 ^b^	13.5 ^b^	12.2 ^b^	−22.6 ^d^	26.8 ^a^	7.5	2.0	224	<0.001
Mn AA	5.5 ^bc^	−9.4 ^d^	18.0 ^a^	−3.6 ^cd^	9.3 ^ab^	9.5 ^ab^	18.0 ^a^	11.1 ^ab^	8.5 ^ab^	7.1 ^b^	7.5	1.4	115	<0.001
Zn AA	28.2 ^c^	9.4 ^d^	36.7 ^abc^	42.7 ^a^	40.9 ^ab^	36.4 ^abc^	37.8 ^ab^	40.2 ^ab^	32.0 ^bc^	33.4 ^bc^	33.7	1.5	28.5	<0.001

^1^ Values represent the means ± standard deviations (SDs) of 5–7 replicate cages of 4 birds per replicate cage (*n* = 5–7). ^a, b, c, d, e^ Means with different superscripts within the same row differ significantly (*p* < 0.05). SBM, soybean meal; AMAs, apparent mineral availabilities; AA, apparent availability; CV, coefficient of variation; Ca, calcium; P, phosphorus; Mg, magnesium; K, potassium; Cu, copper; Fe, iron; Mn, manganese; Zn, zinc.

**Table 7:** SMAs of different sources of SBMs in broilers ^1^.

Items (%)	SBM Numbers										Mean	Pooled SE	CV (%)	*p*-Value
	SBM1	SBM2	SBM3	SBM4	SBM5	SBM6	SBM7	SBM8	SBM9	SBM10				
Ca SA	68.4 ^a^	56.4 ^b^	69.2 ^a^	57.8 ^b^	69.2 ^a^	68.2 ^a^	69.2 ^a^	69.8 ^a^	66.7 ^a^	66.9 ^a^	66.2	0.9	7.4	<0.001
P SA	62.5 ^a^	40.9 ^b^	59.2 ^a^	40.3 ^b^	55.0 ^a^	68.1 ^a^	60.1 ^a^	61.9 ^a^	58.9 ^a^	57.5 ^a^	56.4	1.6	16.0	<0.001
K SA	46.9 ^ab^	40.2 ^bc^	43.4 ^ab^	34.0 ^c^	48.6 ^ab^	51.1 ^a^	44.7 ^ab^	45.9 ^ab^	45.6 ^ab^	49.4 ^a^	45.0	1.1	11.0	0.013
Mg SA	38.8 ^cd^	43.6 ^abc^	34.3 ^d^	35.0 ^d^	44.8 ^abc^	44.1 ^abc^	42.8 ^bc^	46.1 ^ab^	43.5 ^abc^	48.7 ^a^	42.2	0.8	11.1	<0.001
Cu SA	35.3 ^d^	33.2 ^d^	58.0 ^a^	42.5 ^cd^	46.4 ^bc^	50.5 ^abc^	47.8 ^bc^	52.6 ^ab^	52.2 ^bc^	47.2 ^bc^	46.6	1.4	16.6	<0.001
Fe SA	31.8 ^d^	27.6 ^de^	53.5 ^ab^	21.7 ^e^	32.2 ^d^	40.8 ^c^	46.5 ^bc^	49.0 ^b^	12.4 ^f^	58.0 ^a^	37.4	1.9	39.3	<0.001
Mn SA	30.2 ^abc^	16.9 ^d^	39.1 ^a^	22.0 ^cd^	33.5 ^ab^	28.8 ^bc^	35.3 ^ab^	32.8 ^ab^	32.1 ^abc^	33.1 ^ab^	30.4	1.3	21.4	0.002
Zn SA	40.9 ^bc^	23.1 ^d^	49.0 ^ab^	39.1 ^c^	53.7 ^a^	49.2 ^ab^	50.8 ^a^	53.1 ^a^	45.8 ^abc^	49.3 ^ab^	45.4	1.4	20.2	<0.001

^1^ Values represent the means ± standard deviations (SDs) of 5–7 replicate cages of 4 birds per replicate cage (*n* = 5–7). ^a, b, c, d, e, f^ Means with different superscripts within the same row differ significantly (*p* < 0.05). SBM, soybean meal; SMAs, standardized mineral availabilities; SA, standardized availability; CV, coefficient of variation; Ca, calcium; P, phosphorus; Mg, magnesium; K, potassium; Cu, copper; Fe, iron; Mn, manganese; Zn, zinc.

**Table 8:** The correlation coefficient between chemical properties and broiler SMAs in SBMs (air-dry basis).

Items (%)	CP (%)	EE (%)	CF (%)	NDF (%)	ADF (%)	CA (%)	TI (g/kg)	PA (g/kg)	NFE (%)
Ca SA	0.691 *	−0.117	−0.264	−0.458	−0.696 *	0.485	−0.276	−0.895 *	−0.262
P SA	0.787 *	−0.310	−0.455	−0.567	−0.797 *	0.612	−0.105	−0.795 *	−0.089
K SA	0.664	−0.018	−0.711 *	−0.500	−0.781 *	0.452	−0.100	−0.698 *	0.118
Mg SA	0.314	0.035	−0.369	−0.254	−0.307	0.532	0.199	0.003	−0.083
Cu SA	0.698 *	−0.246	−0.082	−0.486	−0.550	0.549	0.124	−0.382	−0.347
Fe SA	0.513	0.325	−0.094	−0.274	−0.336	0.167	−0.016	−0.613	−0.542
Mn SA	0.618	−0.095	−0.096	−0.401	−0.596	0.396	−0.187	−0.803 *	−0.299
Zn SA	0.499	−0.062	−0.020	−0.192	−0.416	0.501	−0.376	−0.675 *	−0.542

* Represents a significant difference (*p* < 0.05); The SMA values of 9 tested SBMs (*n* = 9) were used for correlation (r) analysis. SBM, soybean meal; SMAs, standardized mineral availabilities; SA, standardized availability; CP, crude protein; EE, ether extract; CF, crude fiber; NDF, neutral detergent fiber; ADF, acid detergent fiber; CA, crude ash; TI, trypsin inhibitor; PA, phytic acid; NFE, nitrogen-free extract; Ca, calcium; P, phosphorus; Mg, magnesium; K, potassium; Cu, copper; Fe, iron; Mn, manganese; Zn, zinc.

**Table 9:** Established prediction models of broiler SMAs in SBMs based on their chemical properties (air-dry basis) ^1^.

Prediction Models	*R* ^2^	RMSE	*p*-Value
Ca SA (%) = 67.33 + 2.97 CA − 2.09 PA	0.964	1.14	<0.001
P SA (%) = 50.87 − 1.31 CF + 6.94 CA − 3.14 PA	0.956	2.53	<0.001
K SA (%) = 51.12 − 1.71 NDF − 4.06 ADF	0.823	2.43	0.006
Cu SA (%) = −166.91 + 4.69 CP	0.487	6.28	0.037
Mn SA (%) = 56.24 − 2.58 PA	0.644	4.35	0.009
Zn SA (%) = 145.24 − 2.80 PA − 2.56 NFE	0.672	6.37	0.035

^1^ The SMA values of 9 tested SBMs (*n* = 9) were used to construct the SMA-predicting models. SBM, soybean meal; SMAs, standardized mineral availabilities; SA, standardized availability; CP, crude protein (%); CA, crude ash (%); CF, crude fiber (%); ADF, acid detergent fiber (%); TI, trypsin inhibitor (g/kg); PA, phytic acid (g/kg); EE, ether extract (%); *R*^2^ = coefficient of determination; RMSE, root mean square error; Ca, calcium; P, phosphorus; K, potassium; Cu, copper; Mn, manganese; Zn, zinc.

**Table 10:** Accuracy evaluation of prediction models for SMAs in SBMs for broilers.

Items (%)	Measured Value of SMA in SBM10 ^1^ (%)	Predicted Value of SMA in SBM10 (%)	Difference Value ^2^ (%)	Accuracy Evaluation
Ca SA	66.9 ± 7.3	60.3	6.6	Accurate
P SA	57.5 ± 10.2	50.8	6.7	Accurate
K SA	49.4 ± 6.1	49.6	0.2	Accurate
Cu SA	47.2 ± 8.0	53.9	6.7	Accurate
Mn SA	33.1 ± 8.2	20.9	12.2	Inaccurate
Zn SA	49.3 ± 9.9	32.6	16.7	Inaccurate

^1^ The measured value is the mean ± standard deviation (SD) of 5–7 replicate cages (*n* = 5–7). ^2^ The difference value equals the deviation between measured and predicted values. SBM, soybean meal; SMAs, standardized mineral availabilities; SA, standardized availability; Ca, calcium; P, phosphorus; K, potassium; Cu, copper; Mn, manganese; Zn, zinc.

## Data Availability

All data supporting the findings are included within the article. Additional requests should be addressed to the corresponding author.
